# Inducement and identification of chromosome introgression and translocation of *Gossypium australe* on *Gossypium hirsutum*

**DOI:** 10.1186/s12864-017-4398-7

**Published:** 2018-01-04

**Authors:** Yingying Wang, Shouli Feng, Sai Li, Dong Tang, Yu Chen, Yu Chen, Baoliang Zhou

**Affiliations:** 1 State Key Laboratory of Crop Genetics & Germplasm Enhancement, MOE Hybrid Cotton R&D Engineering Research Center, Nanjing Agricultural University, Nanjing, 210095 Jiangsu People’s Republic of China; 2 Key Laboratory of Cotton Breeding and Cultivation in Huang-Huai-Hai Plain, Ministry of Agriculture, Cotton Research Center of Shandong Academy of Agricultural Sciences, Jinan, 250100 Shandong People’s Republic of China; 3 Institute of Botany, Jiangsu Province and Chinese Academy of Sciences, Nanjing, 210014 China

**Keywords:** Chromosome translocation, Chromosome introgression, Genomic in situ hybridization, *Gossypium hirsutum*, *Gossypium Australe*, Microsatellite marker

## Abstract

**Background:**

We previously reported the development of a set of *Gossypium hirsutum-G. australe* alien chromosome addition lines. Naturally, however, *G. hirsutum*-*G. australe* chromosome exchanges were very limited, impeding the stable transference of useful genes from *G. australe* (G_2_G_2_ genome) into the most cultivated cotton, *G. hirsutum* (AADD).

**Results:**

In the present report, the pollen from a pentaploid (2n = AADDG_2_) of *G. hirsutum-G. australe* was irradiated with seven different doses ranging from 10 to 40 Grays and used to pollinate emasculated flowers of *G. hirsutum* over three consecutive years. Irradiation greatly increased the genetic recombination rates of the *G. hirsutum* and *G. australe* chromosomes and a total of 107 chromosome introgression individuals in 192 GISH-negative (with no GISH signal on chromosome) survived individuals, 11 chromosome translocation individuals (containing 12 chromosome translocation events) and 67 chromosome addition individuals were obtained in 70 GISH-positive (with GISH signal(s) on chromosome(s)) survived individuals, which are invaluable for mining desirable genes from *G. australe*. Multicolor genomic in situ hybridization results showed that there were three types of translocation, whole arm translocation, large alien segment translocation and small alien segment translocation, and that all translocations occurred between the G_2_-genome and the A-subgenome chromosomes in *G. hirsutum*. We also found that higher doses induced much higher rates of chromosome variation but also greatly lowered the seed viability and seedling survivability.

**Conclusions:**

Irradiation has been successfully employed to induce chromosome introgressions and chromosome translocations and promote chromosome exchanges between cultivated and wild species. In addition, by balancing the rates of chromosome introgression and translocation to those of seed set, seed germination, and seedling rates in the M1 generation, we conclude that the dosage of 20 Grays is the most suitable. The established methodology may guide the utilization of the tertiary gene pool of *Gossypium* species such as *G. australe* in cotton breeding in the future.

**Electronic supplementary material:**

The online version of this article (10.1186/s12864-017-4398-7) contains supplementary material, which is available to authorized users.

## Background

*Gossypium australe* F. Mueller, a G_2_ genome diploid species, has numerous agronomically valuable characteristics such as tolerance to abiotic stresses and resistance to insect pests and diseases. *G. australe* (G_2_G_2_) can be used as a donor parent for the genetic improvement of the most important tetraploid cultivated species, *G. hirsutum* (AADD).

Unfortunately, *G. australe*, a species of the tertiary gene pool of *Gossypium*, is a distant relative to *G. hirsutum,* implying scarcities in chromosome pairing and genetic recombination between them. The difficulties with transferring useful genes from *G. australe* into *G. hirsutum* by conventional methods have been validated by previous reports [1, 2]. To transfer favorable genes of interest from species of the tertiary gene pool of *Gossypium*, such as G genome species, the great challenge is how to promote chromosome pairing and genetic recombination. Two main methods have been attempted previously in cotton. The first one is direct backcross method, which has been used by several researchers [3–7]. In the backcross progenies, however, they often observed that whole alien chromosomes were added in the recipient genome and very few chromosomal segment introgressions were found. For example, using two amphidiploids of *G. hirsutum* × *G. australe* and *G. hirsutum* × *G. sturtianum* backcrossed with *G. hirsutum*, Becerra Lopez-Lavalle and Brubaker [4] found that some chromosomes were preferentially eliminated while others were preferentially transmitted and no donor chromatin were clearly introgressed into the recipient *G. hirsutum* genome. Another way is using tri-species hybrid. Zhu et al. [8] made the cross of the amphidiploid F1 of (*G. arboreum* × *G. bickii*) (2n = 4× = 52, A_2_A_2_G_1_G_1_) with *G. hirsutum* and found that the hybrid plants were highly sterile and their chromosome configuration at meiosis metaphase I was 2n = 52 = 41.04 I + 4.54 II + 0.57 III + 0.04 IV, which also demonstrated the low rate of chromosome pairing and genetic recombination between *G. hirsutum* and F1 of (*G. arboreum* × *G. bickii*). Using *G. sturtianum* (2n = 2× = 26, C_1_C_1_) as donor and *G. thurberi* (2n = 2× = 26, D_1_D_1_) or *G. raimondii* (2n = 2× = 26, D_5_D_5_) as bridge species, Vroh Bi et al. [9, 10] synthesized two tri-species hybrids *G. thurberi*–*G. sturtianum*–*G. hirsutum* (TSH) and *G. hirsutum*–*G. raimondii*– *G. sturtianum* (HRS). Their chromosome configurations at metaphase I were 2n = 52 = 15.07I + 15.34 II + 0.93III + 0.69IV + 0.26VI in TSH and 2n = 52 = 14.42I + 17.03 II + 0.82III + 0.15IV + 0.07VI in HRS respectively, indicating that the low rate of chromosome pairings between *G. hirsutum* and *G. sturtianum* (C genome chromosome). Therefore, the low rate of genetic recombination hampered the usage of wild species of the tertiary gene pool in cotton breeding.

Although several methodologies have been used for the inducement of chromosomal exchanges in wheat, such as ionizing irradiation [11], gametocidal chromosomes [12], and pairing homoeologous (*ph*) mutant genes [13, 14], to our knowledge, no gametocidal chromosome or *ph* gene has been found in cotton. Irradiation strategies have therefore become the sole method of promoting the genetic recombination of chromosomes between *G. australe* and *G. hirsutum* for the introgression of favorable genes into *G. hirsutum* from *G. australe*.

Irradiation often induces chromosome breaks. The broken chromosomes then mistakenly rejoin, leading to the production of chromosome aberrations such as translocation and nonhomologous recombination. Irradiation has therefore long been employed to overcome barriers to genetic recombination. For example, the U-genome chromosomes of *Aegilops umbellulata* did not pair with the A-, B- or D-genome chromosomes of wheat (*Triticum aestivum L*., 2n = 6× = 42, AABBDD) during meiosis. To transfer genes of interest from *Aegilops umbellulata* (2n = 2× = 14, UU) to wheat, Sears [11] successfully induced translocation of T6B.6 U between wheat and the U-genome chromosomes and produced a small amount of alien chromatin introgression using irradiation. The irradiation mutagenesis strategy has also been successfully employed to induce nonhomologous chromosome exchanges (i.e., translocation) in upland cotton. To our knowledge, 62 chromosome translocation lines have been identified in *G. hirsutum*, involving 25 of 26 chromosomes, comprising 26 A-A, 10 D-D and 26 A-D chromosome translocations. Fifty four of the 62 translocation lines were induced by irradiation [15]. However, up to date, there are no reports on chromosome translocation and introgression induced by irradiation between tetraploid cultivated and diploid wild cotton.

In this paper, we irradiated pollen from the *G. hirsutum*-*G. australe* pentaploid with ^60^Co-γ rays at several different doses to induce chromosome translocation/introgression and to increase genetic recombination between *G. hirsutum* and *G. australe* chromosomes. Our aims were as follows: (1) to determine a suitable irradiation dose for the production of chromosome translocation or chromosome introgression in cotton distant hybridization breeding; (2) to construct a chromosome mutant library of *G. hirsutum*-*G. australe* for future genomic research on *G. australe*; (3) to establish a methodology for the utilization of the tertiary gene pool of *Gossypium* species such as *G. australe* in cotton breeding.

## Results

### Effects of irradiation dosage on boll set and seed germination

^60^Co γ-ray irradiated pollen from the pentaploid of *G. hirsutum*-*G. australe* (2n = 5X = AADDG = 65) was pollinated to the emasculated flowers to produce hybrid seeds.

In 2011, preliminary pollen irradiation with three doses (10, 12, and 20 Grays, Gy) was performed. The results indicated that all the boll set rates were >80% and plenty of seeds were obtained from irradiation treatments, implying these three irradiation doses had little influence on boll set. We then planted the seeds and found that as the dose increased, the germination rates and number of seedlings decreased. Even so, we concluded that a dose of 20 Gy or higher is suitable since the germination rates were up to 44.85% and 14.71% of seedlings were generated at the dose of 20 Gy (Table 1).

**Table 1:** Boll setting and seed germination in the M_1_ induced by irradiation at different doses

Year	Dose (Gy)	Flowers pollinated	Bolls set	Boll set (%)	Seeds obtained	Seeds per boll	Seeds sown	Germinated seeds	Germination (%)	Seedlings	Seedling (%)
2011	10	362	331	91.44	1016	2.81	226	158	69.91	47	20.80
	12	277	271	97.83	234	0.84	234	153	65.38	42	17.95
	20	92	75	81.52	136	1.48	136	61	44.85	20	14.71
2012	0 (CK)	135	132	97.78	129	0.96	129	120	93.02	102	79.07
	20	391	149	38.11	385	0.98	385	122	31.69	20	5.19
	30	229	114	49.78	189	0.83	189	58	30.69	8	4.23
	40	123	35	28.46	35	0.28	35	8	22.86	1	2.86
2013	15	395	337	85.32	125	0.32	125	44	35.20	34	27.20
	20	511	423	82.78	118	0.23	118	28	23.73	20	16.95
	25	234	193	82.48	6	0.03	6	0	0.00	0	0.00

Based on these initial results, in 2012, higher doses of irradiation (20, 30, and 40 Gy) were employed. The results showed that boll set rates were significantly decreased to the half of the control, and seed germination rates and seedling percentages greatly dropped, reaching 22.86-31.69% and 2.86-5.19%, respectively. The results implied that doses of 30 and 40 Gy are too high for pollen irradiation. In 2013, the doses were further adjusted to 15, 20, and 25 Gy. To obtain much more M1 progenies, much more flowers were pollinated by irradiated pollen to produce more hybrid seeds. However, most of the obtained seeds were immature and were of the lower viability because cotton plants suffered from low temperature and frost during the late development. Therefore, cotton seeds harvested in 2013 showed very low rates of germination and viability. The results demonstrated that all the boll set rates were >80%, while only at the lower two doses were slightly more seeds generated and 23.73-35.20% of seeds germinated and produced 16.95-27.20% of seedlings. At the dose of 25 Gy only six seeds were produced and did not germinate (Table 1).

On account of the effects of seven doses from 10 to 40 Gy in 3 years on boll set rates and the obtained seed vigor, we presumed that doses of 15 or 20 Gy were suitable for pollen irradiation treatment, since higher doses led to lower boll set rates and further detrimental effects on seed vigor.

### Discrimination of chromosome aberrations by genomic in situ hybridization (GISH)

**Fig. 1:**
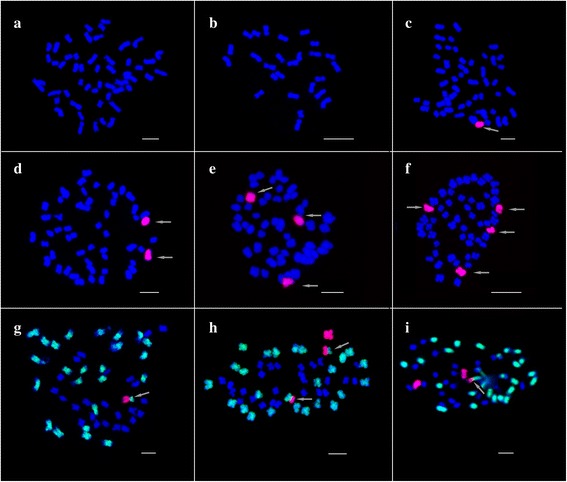
GISH analysis of chromosome components in the progenies of the *Gossypium hirsutum*-*G. australe* pentaploid (2n = AADDG_2_) pollinated by irradiation-induced pollen. **a.** Mitotic chromosome spread of the 52 chromosomes of *G. hirsutum*. **b.** Mitotic chromosome spread of the 26 chromosomes of *G. australe*. **c**- **f.** Mitotic chromosome spread showing the 52 *G. hirsutum* chromosomes (blue signals) plus one (1G), two (5G, 8G), three (3G, 9G, 12G) and four (6G, 7G, 8G, 9G) chromosomes of *G. australe* (red signals, white arrowhead). **g-i.** GISH revealing the *G. hirsutum-G. australe* translocation chromosomes (white arrowhead). The gDNA of *G. australe* was labeled with digoxigenin-11-dUTP and chromosomes from *G. australe* were visualized as red signals; the gDNA of *G. herbaceum* was labeled with biotin-16-dUTP and the resultant A-subgenome chromosomes in *G. hirsutum* were visualized as green signals; D-subgenome chromosomes in *G. hirsutum* were counterstained with 4, 6-diamidino-2-phenylindole (DAPI) and were visualized as blue signals

Between 2011 and 2013, a total of 632 M_1_ generation individuals were analyzed by GISH. Among them, 170 GISH-positive (with GISH signal(s) on chromosome(s)) individuals were identified and 11 groups of chromosome aberrations were characterized, namely, four groups of alien chromosome additions that contain one to four alien chromosomes (142 in 170 GISH-positive individuals), one group of sole translocations (10 in 170 GISH-positive individuals), and six groups of both alien chromosome additions and translocations (18 in 170 GISH-positive individuals) (Table 2; Fig. 1). In the year 2012, only 13 control individuals were identified as GISH-positive from 120 individuals and one group was characterized, and found to contain monosomic alien chromosome additions. Therefore, pollen irradiation can effectively induce alien chromosome aberrations.

**Table 2:** Chromosomal variants induced by irradiation at different doses

Year	Dose (Gy)	Seeds germinated	GISH positive	GISH (%)	Groups of variants	1 Add^a^	2 Add	3 Add	4 Add	1 Tr^b^	1 Tr + 1 Add	2 Tr + 1 Add	1Tr + 2 Add	2Tr + 2 Add	1Tr + 3 Add	2Tr + 4 Add	Total of Tr	Tr (%)
2011	10	158	32	20.92	6	17	8	4	1	1	0	0	1	0	0	0	2	1.27
	12	153	50	31.65	6	33	8	2	0	4	1	0	2	0	0	0	7	4.58
	20	61	21	34.43	6	11	2	0	0	2	3	0	2	1	0	0	8	13.11
2012	0(CK)	120	13	10.83	1	13	0	0	0	0	0	0	0	0	0	0	0	0.00
	20	122	31	25.41	9	18	2	3	0	3	1	1	1	1	1	0	8	6.56
	30	58	15	25.86	5	10	2	1	1	0	0	0	1	0	0	0	1	1.72
	40	8	4	50.00	3	2	0	0	0	0	1	0	0	0	0	1	2	25.00
2013	15	44	10	22.73	2	8	2	0	0	0	0	0	0	0	0	0	0	0.00
	20	28	7	25.00	1	7	0	0	0	0	0	0	0	0	0	0	0	0.00
	25	0	0	–	0	0	0	0	0	0	0	0	0	0	0	0	0	–
Total		632	170	26.90	11	106	24	10	2	10	6	1	7	2	1	1	28	4.43

^a^,chromosomes added; ^b^, chromosome translocation

In the M_1_ generation, most aberrations in GISH-positive individuals were monosomic alien chromosome additions (106/170), followed by double monosomic alien chromosome additions (24/170), triple monosomic alien additions (10/170) and one chromosome translocations (10/170), one chromosome translocations plus double monosomic alien additions (7/170), and one chromosome translocations plus monosomic alien additions (6/170). The other five groups of aberrations (quadruple monosomic alien additions, double chromosome translocations plus double monosomic alien additions, double chromosome translocations plus monosomic alien additions, double chromosome translocations plus quadruple monosomic alien additions, and one chromosome translocation plus triple monosomic alien additions) were scarce in GISH-positive lines (Table 2; Fig. 1).

**Fig. 2:**
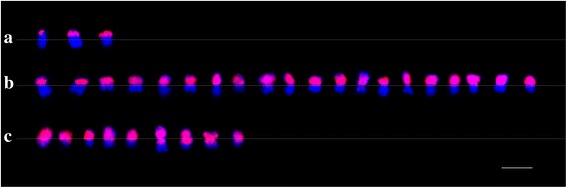
Chromosome translocations revealed by GISH. **a.** small alien segment translocation. **b**. whole arm translocations. **c**. large alien segment translocations

Moreover, among the 28 individuals containing translocations, we found that 32 breakage-fusion events had occurred, because two translocations each were present in 4 of the 28. Among them, 11 individuals containing 12 translocation events survived. Based on the sizes and inserted positions of the alien chromosomes, translocation could generally be divided into five types, namely, whole arm translocation (WAT), terminal translocation (TT), large alien segment translocation (LAST), small alien segment translocation (SAST), and intercalary translocation with the inserted segment from *G. australe* (IT). Here, three types of translocation were discovered, most of them being WAT (22/32), followed by LAST (7/32), and SAST (3/32) (Fig. 2). The other two types of translocations (TT and IT) were not found.

Between 2011 and 2013, seven doses were attempted to find a suitable dose for the inducement of chromosome mutations, of which only 20 Gy was used in all 3 years. Our results demonstrated that different doses produced different effects on chromosome variation and the same dose (like 20 Gy) generated significantly different effects in different years as well. Even so, it is not difficult to conclude that the dose of 20 Gy was a suitable dose for the induction of chromosome mutation due to the abundance of chromosome variations it induced.

### Incidence of alien (*G. australe*) chromosome aberrations on M_1_ generations as identified by molecular marker analysis

Seventy GISH-positive individuals survived and the others died due to lower viability resulting from chromosome variations. Among the 70 surviving individuals, 59 contained chromosome additions only, 3 contained chromosome translocations only, and 8 contained both chromosome additions and chromosome translocations. *G. australe*-specific simple sequence repeat (SSR) markers screened by our previous report [5] were employed to determine the identity of the *G. australe* chromosomes in the *G. hirsutum* background of the M_1_ generation. The results indicated that among the 67 individuals with alien chromosomes added, chromosomes 5G, 6G and 8G had the greatest incidence (22.39%), followed by 12G (17.91%), 9G (16.42%), 2G (7.46%) and 11G (7.46%), 7G (5.97%), 3G (4.48%), 4G (4.48%), 10G (4.48%), and 1G (2.99%). No incidence of added chromosome 13G was found. Among the 11 individuals with chromosome translocations, there were two incidences each of 3G, 7G, 8G, and 13G translocations, and one of 2G, 6G, 9G, and 10G translocations. No translocations of the other five chromosomes (1G, 4G, 5G, 11G, and 12G) were found (Table 3; Fig. 1g-i). Three single chromosome translocations of chromosomes 2G, 7G, and 8G were found at very low incidences (1/70) in GISH-positive individuals and at the extremely low incidences (1/632) in the M_1_ population (Additional file 1**)**.

**Table 3:** Incidence of chromosomal variants in *G. hirsutum* × *G. australe* in the M_1_ generation

Chromosome	1G	2G	3G	4G	5G	6G	7G	8G	9G	10G	11G	12G	13G	Individuals
52 + 1 Add	1	3	2	1	7	7	2	5	3	3	4	6	0	44
52 + 2 Add	1	0	0	0	5	3	0	6	3	0	1	5	0	12
52 + 3 Add	0	0	1	0	0	0	0	0	1	0	0	1	0	1
52 + 4 Add	0	0	0	1	1	2	1	1	2	0	0	0	0	2
2n(1 Tr.)	0	1	0	0	0	0	1	1	0	0	0	0	0	3
52(1Tr) + 1 Add	0	0	1^a^	0	1^b^	1^b^	1^a^	0	1^b^	0	0	0	1^a^	3
52(1Tr) + 2 Add	0	2^b^	1^a^	1^b^	0	2^b^ + 1^a^	0	2^b^ + 1^a^	1^b^ + 1^a^	0	0	0	0	4
52(2Tr) + 2 Add	0	0	0	0	1^b^	0	0	1^b^	0	1^a^	0	0	1^a^	1
Chromosome Add^c^	2	5	3	3	15	15	4	15	11	3	5	12	0	67
Incidence of Add (%)	2.99	7.46	4.48	4.48	22.39	22.39	5.97	22.39	16.42	4.48	7.46	17.91	0.00	100.00
Chromosome Tr^d^	0	1	2	0	0	1	2	2	1	1	0	0	2	11
Incidence of Tr (%)	0.00	9.09	18.18	0.00	0.00	9.09	18.18	18.18	9.09	9.09	0.00	0.00	18.18	100.00
52 + 1 Add												13		13
Incidence of Add (%)												100.00		100.00

^a^, chromosomes translocated; ^b^, chromosomes added; ^c^, chromosomes added; ^d^, chromosomes translocated

### Identification of the *G. hirsutum* chromosomes translocated by the chromosomes from *G. australe*

To further determine the identity of the *G. hirsutum* chromosomes involved in translocation, we analyzed somatic cells of all the 28 translocation lines by multicolor-GISH using the total genomic DNA of *G. australe* and *G. herbaceum* as probes, and *G. raimondii* as a blocker (see Materials and methods). Using this technique, the G- and A-subgenome chromosomes would be revealed as red and yellow signals, respectively; and the D-subgenome chromosomes would produce blue signals. The multicolor-GISH results showed that all translocations occurred between chromosomes of the A-subgenome in *G. hirsutum* and those of *G. australe* (Fig. 1g-i) and no translocations occurred between chromosomes of the D-subgenome in *G. hirsutum* and those of *G. australe*.

### Chromosomal segment introgression from *G. australe* into *G. hirsutum* induced by irradiation

Between 2011 and 2013, in the M_1_ generation, a total of 192 GISH-negative (with no GISH signal on chromosome) individuals were analyzed using SSR markers that are evenly distributed on the D-subgenome chromosomes of tetraploid cotton. The results indicated that 107 out of 192 individuals were introgressed, each by a number of alien chromosomes ranging from one to five, of which most of individuals (71/192) were introgressed by one alien chromosome, followed by two (18/192), three (14/192), four (3/192), and five (1/192) (Table 4 and Additional file 2). The other 85 individuals were not introgressed by any alien chromosomes. The average introgression percentage in the M_1_ generation was 55.73% (107/192), which was far higher than that in controls (11.76%). In the control (no irradiation), 102 individuals were analyzed using the SSR markers. Only 12 plants were found to be introgressed by alien chromosomes. Among them, nine plants were each introgressed by two alien chromosomes, followed by one (2/12) and three (1/12). The results also showed that the percentages of introgression were increased with the increasing irradiation dose, except in 2011 (Additional file 2).

**Table 4:** Identification of alien chromosomal segment introgression in the M_1_ generation

	chromosome	1G	2G	3G	4G	5G	6G	7G	8G	9G	10G	11G	12G	13G	No. individuals
Irradiated	0														85
	1	5	4	0	1	21	8	0	12	8	5	5	1	1	71
	2	5	3	0	4	10	3	0	3	2	1	1	2	2	18
	3	7	4	1	4	6	4	0	2	1	7	2	1	3	14
	4	1	2	0	1	2	0	0	0	0	2	1	1	2	3
	5	1	1	0	1	1	0	0	0	0	0	0	0	1	1
	Sum	19	14	1	11	40	15	0	17	11	15	9	5	9	107
	Introgression (%)	9.90	7.29	0.52	5.73	20.83	7.81	0.00	8.85	5.73	7.81	4.69	2.60	4.69	55.73
CK	0														90
	1	0	2	0	0	0	0	0	0	0	0	0	0	0	2
	2	0	0	0	0	9	0	0	0	0	0	0	0	9	9
	3	0	0	0	0	1	0	0	0	1	0	0	0	1	1
	Sum	0	2	0	0	10	0	0	0	1	0	0	0	10	12
	Introgression (%)	0.00	1.96	0.00	0.00	9.80	0.00	0.00	0.00	0.98	0.00	0.00	0.00	9.80	11.76

In the M_1_ generation, the identities of the introgressive chromosomal segments were analyzed using SSR markers. Our results showed that the most frequent introgressive chromosomal segments caused by the irradiation were from chromosome 5G (20.83%), followed by 1G (9.90%), 8G (8.85%), 6G and 10G (both 7.81%), 2G (7.29%), 9G and 4G (both 5.73%), 11G and 13G (4.69%), and 3G (0.52%). Chromosome 7G did not give rise to any introgressive chromosomal segments following irradiation. In control plants, however, only four chromosomes were found to be introgressed. Chromosomes 5G and 13G were simultaneously introgressed at a rate of 9.80%, 2G at a rate of 1.96% and 9G at 0.98%. No other chromosomes were found to be introgressed (Table 4). Therefore, it is concluded that chromosome introgression in cotton distant hybridization can be induced or enhanced by pollen irradiation.

## Discussion

### It is important to determine a suitable irradiation dose for the enhancement of genetic recombination in distant hybridization breeding

Irradiation often can be used to induce chromosome breakage and fusion events, to generate numerous chromosomal variants, to obtain progenies from nonhomologous chromosome exchanges, and to give rise to increased genetic recombination in distant hybridization breeding [16]. Cotton irradiation mutagenesis is generally seed-based, but the irradiated seed grows into chimeras. Pollen contains sperm cells that are sensitive to irradiation mutagenesis. Mutations in sperm can be passed on to offspring and help to improve the efficiency of selection and accelerate the process of irradiation breeding. ^60^C_O_-γ rays are the most widely used mutagens in cotton breeding.

In this study, to induce chromosomal exchanges between *G. australe* and *G. hirsutum*, we used seven doses of ^60^C_O_-γ rays, ranging from 10 to 40 Gy, to irradiate pollen from the pentaploid of *G. hirsutum* × *G. australe*. Our results demonstrated that higher doses not only induced much higher rates of variations but also greatly lowered the seed viability and the seedling survivability. Therefore, it is presumed that a dose of 20 Gy is suitable for pollen irradiation.

### A preliminary *G. hirsutum*-*G. australe* chromosome mutant library has been constructed for genomic research on *G. australe*

Several types of chromosome libraries can be constructed from interspecific hybridization data, such as substitution lines, introgression lines, chromosome alien addition lines, and chromosome translocation lines. Previously, only three incomplete sets of cotton chromosome substitution lines, between *G. hirsutum* and *G. barbadense*, *G. mustelinum*, and *G. tomentosum,* had been developed using aneuploidy as a tool [17–19]. Several sets of cotton introgression lines (also known as chromosome segment substitution lines) were also developed between *G. hirsutum* and *G. barbadense* [20–23], and *G. mustelinum* [24]. These two types of line were previously limited to species with the same ploidy level and the same genome components (i.e. those belonging to the same primary gene pool).

However, no sets of diploid species have previously been chromosome substituted or chromosome introgressed into tetraploid cotton. In this study, 107 individuals involving 12 out of 13 introgressive chromosomes were obtained at the small chromosome segment level. Of them, 71 were introgressed in the *G. hirsutum* background by single chromosomes, and this is invaluable for mining desirable genes that are unavailable in cultivated cotton species.

Moreover, 11 chromosome translocations were found; three of which that involved one chromosome each (2G, 7G, and 8G) have not been reported before in cotton, despite numerous reports in wheat where they allowed the wheat breeders to extensively transfer useful genes from wild relatives into common wheat [14, 25, 26].

In addition, it is necessary to point out that until now, only two sets of chromosome alien addition lines in cotton had been developed by our lab [5, 27]. Here, from the M_1_ generation, we also found that 44 individuals were monosomic alien additions that involved 12 chromosomes (all except chromosome 13G). Of them, the chromosome 7G addition was first isolated in our lab.

### Establishment of a methodology for the utilization of the tertiary gene pool of *Gossypium* species such as *G. australe* in cotton breeding

Several obstacles exist in the process of cotton distant hybridization breeding using wild relatives, such as interspecific cross incompatibility, interspecific hybrid F_1_ sterility, low rates of genetic recombination giving rise to linkage drag, and challenges in the reliable identification and characterization of the progenies of interspecific hybridization in mitotic and meiotic cells due to the small size of chromosomes in cotton (2n = 52). Our previous reports showed that the interspecific cross incompatibility can be alleviated via embryo rescue and F_1_ sterility can be overcome by chromosome doubling through treatment with colchicines [28]. Identification and characterization of alien chromosomes in mitotic and meiotic cells of the progenies of interspecific hybridization can be easily achieved through a combination of GISH and molecular marker (SSR) analysis [5, 29, 30]. GISH allows us to understand the numbers of alien chromosomes/segments, and markers help us to discriminate the identity of alien chromosomes/segments. However, there are few reports on how to enhance genetic recombination and promote chromosome exchange between chromosomes of cultivated and wild cotton, especially in tertiary gene pool species, and how to reduce or break the linkage drag between favorable and unfavorable genes.

Irradiation often induces chromosome breakages and fusions and increases rates of genetic recombination. In this report, an attempt to induce exchanges between chromosomes of cultivated tetraploid cotton and wild diploid species (belonging to the tertiary gene pool) and to enhance genetic recombination was made. Several doses of ^60^Co -γ rays, ranging from 10 to 40 Gy, were employed to irradiate pollen from an interspecific pentaploid hybrid (2n = 5X = AADDG_2_ = 65) of *G. hirsutum* × *G. australe* to induce chromosome exchanges and enhance chromosomal segment introgression from wild species. The results indicated that irradiation greatly increased genetic recombination rates and a total of 107 chromosome introgressions and 28 chromosome translocations were obtained, which will be invaluable for mining desirable genes from *G. australe* in the future. On balancing the rates of chromosome introgression and chromosome translocation to those of seed set, seed germination, and seedling survival in the M_1_ generation, we found that the dose of 20 Gy was the most suitable.

We are therefore able to summarize a methodology for the utilization of the tertiary gene pool of a *Gossypium* species such as *G. australe* in cotton breeding in the follow four steps. Interspecific cross incompatibility, firstly, can be alleviated via embryo rescue. Secondly, F_1_ sterility can be overcome by chromosome doubling through treatment with colchicines. Thirdly, linkage drags and a lack of genetic recombination in interspecific hybrids between cultivated and wild species can be alleviated by irradiation. Finally, identification and characterization of alien chromosomes or segments in the progenies of interspecific hybridization can be performed through a combination of GISH and molecular marker (SSR) analysis. Based on our data, it can be concluded that this methodology will facilitate the use of wild species (especially tertiary pool species) in breeding and the exploitation of more favorable genes from wild relatives.

## Conclusions

In this work, we show that irradiation can be used to induce chromosome introgressions and chromosome translocations and to promote chromosome exchanges between cultivated and wild species, which are invaluable for mining genes of interest from *G. australe*. We also demonstrate that higher doses induced much higher rates of chromosome variation but also greatly lowered the seed viability and seedling survivability. By balancing the rates of chromosome introgression and translocation to those of seed set, seed germination, and seedling rates in the M1 generation, we conclude that the dosage of 20 Grays is the most suitable. The established methodology may guide the utilization of the tertiary gene pool of *Gossypium* species such as *G. australe* in cotton breeding in the future.

## Methods

### Plant materials

Four pentaploid plants obtained through the hybridization of an allohexaploid of *Gossypium hirsutum* -*G. australe*) [5] and *G. hirsutum* acc CL-2, were grown at Pailou Breeding Station of Nanjing Agricultural University. The allohexaploid was kindly provided by Dr. Brubaker. CL-2 has a high boll-setting and big boll size characteristic, which easily produce hybrid seeds when used as a parent for crossing. In this study, CL-2 as the maternal parent was pollinated with irradiated pollens from the pentaploid plants as the paternal parent.

### Irradiation treatments

Fresh pollen collected from the four pentaploid plants at anthesis was irradiated with ^60^Co γ-rays (10, 12 and 20 Grays (Gy) in 2011; 20, 30 and 40 Gy in 2012; 15, 20 and 25 Gy in 2013) at a dosage rate of 1.0 Gy/min at the Institute of Atomic Energy, Jiangsu Academy of Agricultural Sciences. Fresh irradiated pollen was pollinated to the female parent CL-2 that was emasculated the day before. The plant hormone, gibberellin (GA_3_) (50 mgL^−1^, *w*/*v*), was dropped to the flower base once a day for seven consecutive days to protect against shedding after pollination. Hybrid seeds were harvested and used to produce M_1_ populations. Pollen collected from four untreated pentaploid plants was also used to pollinate CL-2 as a control.

For each irradiation dose, boll set rate, F_1_ seed number, seed numbers per boll, F_1_ seed germination percentage, F_1_ seedlings, and F_1_ plant chromosome aberration were surveyed.

### Chromosome preparation

Cotton root tips were used to prepare chromosomes by the conventional squashing method with some modifications [5]. Root tips from each F_1_ individual were collected from germinated seeds (roots 3 cm long) and pretreated in 25 μg/ml cycloheximide at room temperature for 2 h to accumulate metaphase cells, then fixed in methanol-acetic acid (3:1) fixative and stored in 70% *v*/v ethanol. After fixation, root tips were washed in distilled water and then macerated in a mixture of 2% cellulose and 0.5% pectolyase at 37 °C for 0.5 h. The mixture were carefully washed from the softened material and replaced with methanol-acetic acid (3:1) fixative. A chromosome spread was prepared as described previously [30]. Cytological observations were performed under a BX51 Olympus phase-contrast microscope (Olympus Corp., Tokyo, Japan). Slides with >20 good images of well-spread chromosomes at metaphase in mitotic cells were prepared and then stored at −70 °C until use. After removing the cover glasses, slides were dehydrated through an ethanol series (70, 90, and 100%; 5 min each). Before use in GISH, slides were immersed in 2 × SSC (saline sodium citrate) containing 100 ng/ml RNase A at 37 °C for 1 h and washed twice with 2 × SSC (37 °C, 5 min each wash).

### DNA probe preparation and molecular marker analysis

Total genomic DNA was extracted from young leaves of *G. hirsutum* (2n = 4X = 52, AADD), *G. australe* (2n = 2X = 26, G_2_G_2_), *G. raimondii* (2n = 2X = 26, D_5_D_5_), *G. herbaceum* (2n = 2X = 26, A_1_A_1_), the interspecific hexaploid (2n = 6X = 78, AADDG_2_G_2_), and individuals of the M_1_ generations, as described by Paterson et al. [31] with some modifications. Genomic DNA of *G. australe* and *G. herbaceum* were labeled with digoxygenin-11-dUTP and biotin-16-dUTP, respectively, by nick translation (Roche, Germany). The sizes of the labeled DNA probe fragments were between 200 and 500 bp. Detection and visualization were performed as described by Han et al. [30].

Based on our previous report [5], a total of 245 *G. australe*-specific SSR marker alleles that were almost evenly distributed on each Dt-subgenome chromosome were selected to characterize the genomic composition in the M_1_ generation (Additional file 3). *G. australe* and the hexaploid of *G. hirsutum*-*G. australe* were used as positive controls, while TM-1 and CL-2 were used as negative controls.

### Genomic in situ hybridization (GISH)

The in situ hybridization protocol was modified slightly from Hanson et al. [32] and Jiang et al. [33]. A hybridization solution was produced as per Guan et al. [34] with some modifications. About 25-50 ng of labeled genomic-DNA was applied to each slide in a hybridization solution with 50% formamide, 10% *w*/*v* dextran sulfate, a suitable amount of sheared cotton DNA as blocking DNA (probe: blocking DNA = 1:100–150), and 2 × SSC. The mixture was denatured at 97 °C for 10 min, chilled on ice, and applied to a dried slide. Slide-bound chromosomal DNA was denatured in a solution of 70% formamide in 2 × SSC for 1.5 min at 70 °C and immediately dehydrated in an ethanol series (70, 90, and 100%; 5 min each) at −20 °C and air-dried. Twenty microliters of hybridization mixture was applied to each slide and sealed under a coverslip (20 × 20 mm) with rubber cement.

After overnight incubation at 37 °C, the coverslips were removed and the slides were washed at 40 °C in 2 × SSC twice for 5 min each, 50% formamide in 2 × SSC for 10 min, 2 × SSC for 5 min, and 1 × PBS for 5 min. Biotin-labeled probes were detected with avidin-fluorescein (green) and dig-labeled probes were detected with anti-digoxygenin-rhodamine (red) (Roche Diagnostics). Following the post hybridization washes, slides were stained with 4,6-diamidino-2-phenylindole (DAPI, Roche Diagnostics) for 5 min at room temperature, and finally, antifade (Vector, USA) was applied under a coverslip. GISH images were captured using an Evolution VF CCD camera (Media Cybernetics, USA) installed on an Olympus BX51 fluorescence microscope, and merged using Image-Pro Express software 5.0 (Media Cybernetics, Bethesda, MD, USA). Final image adjustments were performed using Adobe Photoshop 6.

## Additional files

Additional file 1: Electrophoresis patterns of *G. australe* chromosomes and chromosome fragments specific SSR markers in *G. hirsutum*. From a to o, the *G. australe*-specific primers were NAU5172 (D1), NAU6728 (D2), NAU805 (D3), cgr5566 (D4), JESPR134 (D5), NAU5475 (D5), dPL0702 (D6), NAU2680 (D7), NAU3904 (D8), NAU7616 (D8), NAU3769 (D9), NAU493 (D10), CIR275 (D11), NAU1558 (D12) and NAU3211 (D13). M, DNA ladder; P1, *G. hirsutum*; P2, *G.australe*; F1, the hexaploid of *G. hirsutum* and *G. australe*; Lanes 1 to 45 indicate partial individuals in M1 generation. a and b, plants 5 and 45 carry chromosomal segments of 1G^a^ and 2G^a^, respectively; c, plants 9, 15 and 34 carry chromosomal segments of 3G^a^; d, plants 10 and 33 carry chromosomal segments of 4G^a^; e, plants 10, 18, 26, 38 and 42 carry chromosomal segments of 5G^a^; f, plants 1, 3, 9, 13, 17, 18, 22, 24, 25, 28, 29, 31, 34, 35, 39 and 40 carry chromosomal segments of 5G^a^; g, plants 6, 10, 18, 39 and 43 carry chromosomal segments of 6G^a^; h, plant 8 carries chromosomal segment of 7G^a^; i, plants 2, 11, 25 and 26 carry chromosomal segments of 8G^a^; j, plants 11, 25 and 26 carry chromosomal segments of 8G^a^; k, plants 6, 9, 10, 29 and 36 carry chromosomal segments of 9G^a^; l and m, plants 27 and 21 carry chromosomal segments of 10G^a^ and 11G^a^, respectively; n, plants 9, 13, 19, 24 and 29 carry chromosomal segments of 12G^a^; o, all plants carry no chromosomal segments of *G. australe*. The red arrows demonstrated that the bands were amplified from *G. australe* -specific chromatins. (TIFF 4096 kb)

Additional file 2: Chromosome introgression induced by irradiation at different doses. (DOCX 14 kb)

Additional file 3: The set of SSR markers and their locations on the genome that were used for the identification of *G. australe* chromatins. Note: One hundred and forty pairs of SSR markers in bold (screened by Chen et al. [5]) and One hundred and five pairs of markers in red (screened in this study) were used while twenty pairs of underlined markers (screened by Chen et al. [5]) were not used due to their low reproducibility. The locations of SSRs on the genome are based on the backbone map of the Dt subgenome of tetraploid cotton constructed using the BC1 population of (*G. hirsutum* × *G. barbadense*) × *G. hirsutum* (Guo et al. 2007). (TIFF 10040 kb)

## Abbreviations

DAPI: 4,6-diamidino-2-phenylindole
GA_3_: Gibberellins
GISH: Genomic in situ hybridization
Gy: Gray
IT: Intercalary translocation
LAST: Large alien segment translocation
SAST: Small alien segment translocation
SSR: Simple sequence repeat
TT: Terminal translocation
WAT: Whole arm translocation
