# Supplementary figures and images for: Inducement and identification of chromosome introgression and translocation of Gossypium australe on Gossypium hirsutum
Source: BMC Genomics. 2018 Jan 4;19:15. doi: 10.1186/s12864-017-4398-7 (PMC5755069; doi:10.1186/s12864-017-4398-7)

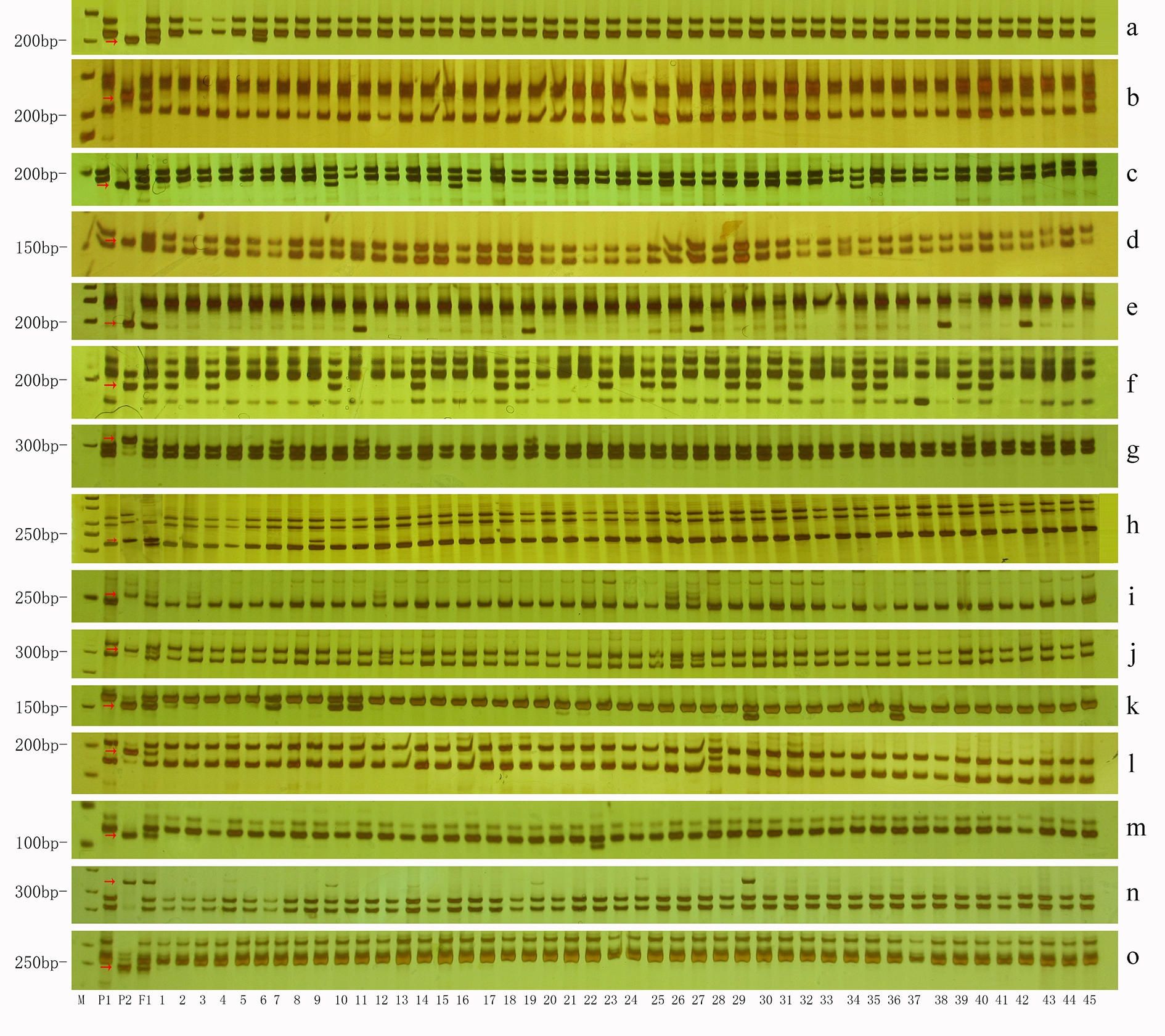

Supplement: Supplementary file 1 — Electrophoresis patterns of G. australe chromosomes and chromosome fragments specific SSR markers in G. hirsutum. From a to o, the G. australe-specific primers were NAU5172 (D1), NAU6728 (D2), NAU805 (D3), cgr5566 (D4), JESPR134 (D5), NAU5475 (D5), dPL0702 (D6), NAU2680 (D7), NAU3904 (D8), NAU7616 (D8), NAU3769 (D9), NAU493 (D10), CIR275 (D11), NAU1558 (D12) and NAU3211 (D13). M, DNA ladder; P1, G. hirsutum; P2, G.australe; F1, the hexaploid of G. hirsutum and G. australe; Lanes 1 to 45 indicate partial individuals in M1 generation. a and b, plants 5 and 45 carry chromosomal segments of 1Ga and 2Ga, respectively; c, plants 9, 15 and 34 carry chromosomal segments of 3Ga; d, plants 10 and 33 carry chromosomal segments of 4Ga; e, plants 10, 18, 26, 38 and 42 carry chromosomal segments of 5Ga; f, plants 1, 3, 9, 13, 17, 18, 22, 24, 25, 28, 29, 31, 34, 35, 39 and 40 carry chromosomal segments of 5Ga; g, plants 6, 10, 18, 39 and 43 carry chromosomal segments of 6Ga; h, plant 8 carries chromosomal segment of 7Ga; i, plants 2, 11, 25 and 26 carry chromosomal segments of 8Ga; j, plants 11, 25 and 26 carry chromosomal segments of 8Ga; k, plants 6, 9, 10, 29 and 36 carry chromosomal segments of 9Ga; l and m, plants 27 and 21 carry chromosomal segments of 10Ga and 11Ga, respectively; n, plants 9, 13, 19, 24 and 29 carry chromosomal segments of 12Ga; o, all plants carry no chromosomal segments of G. australe. The red arrows demonstrated that the bands were amplified from G. australe -specific chromatins. (TIFF 4096 kb) [file 12864_2017_4398_MOESM1_ESM.tif]

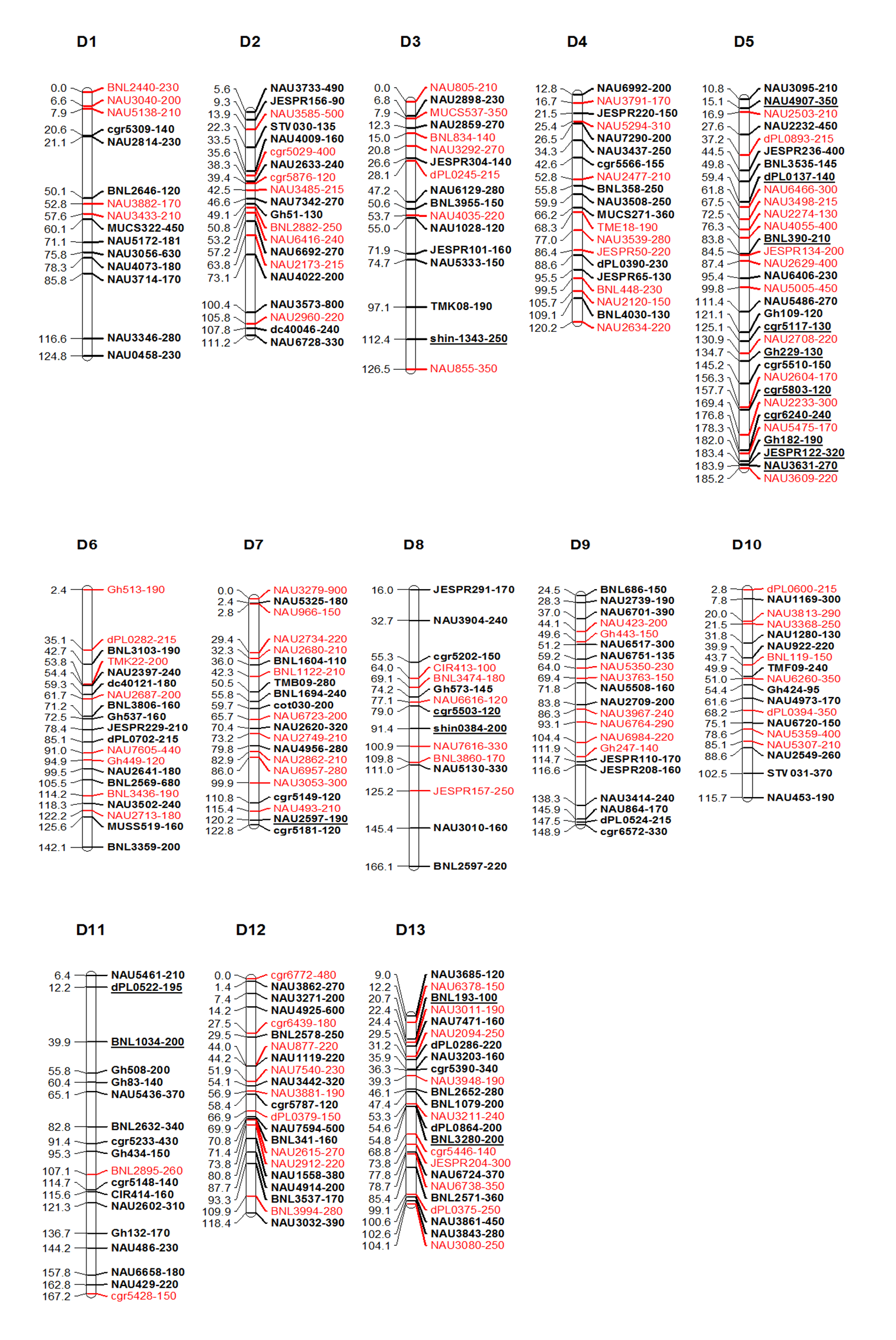

Supplement: Supplementary file 3 — The set of SSR markers and their locations on the genome that were used for the identification of G. australe chromatins. Note: One hundred and forty pairs of SSR markers in bold (screened by Chen et al. [5]) and One hundred and five pairs of markers in red (screened in this study) were used while twenty pairs of underlined markers (screened by Chen et al. [5]) were not used due to their low reproducibility. The locations of SSRs on the genome are based on the backbone map of the Dt subgenome of tetraploid cotton constructed using the BC1 population of (G. hirsutum × G. barbadense) × G. hirsutum (Guo et al. 2007). (TIFF 10040 kb) [file 12864_2017_4398_MOESM3_ESM.tif]
