# Supplementary material for: Inducement and identification of chromosome introgression and translocation of Gossypium australe on Gossypium hirsutum
Source: BMC Genomics. 2018 Jan 4;19:15. doi: 10.1186/s12864-017-4398-7 (PMC5755069; doi:10.1186/s12864-017-4398-7)
Supplement: Supplementary file 2 — Chromosome introgression induced by irradiation at different doses. (DOCX 14 kb) [file 12864_2017_4398_MOESM2_ESM.docx]

**Additional file 2** Chromosome introgression induced by irradiation at different doses

| Year | Dose (Gy) | Seedling | No. alien chromosomes involved | | | | | Introgression | Introgression (%) |
| --- | --- | --- | --- | --- | --- | --- | --- | --- | --- |
|  |  |  | 1 | 2 | 3 | 4 | 5 |  |  |
| 2011 | 10 | 47 | 18 | 6 | 1 | 1 | 0 | 26 | 55.32 |
|  | 12 | 42 | 21 | 7 | 6 | 2 | 0 | 36 | 85.71 |
|  | 20 | 20 | 6 | 3 | 1 | 0 | 0 | 10 | 50.00 |
| 2012 | 0(CK) | 102 | 2 | 9 | 1 | 0 | 0 | 12 | 11.76 |
|  | 20 | 20 | 13 | 0 | 0 | 0 | 0 | 13 | 65.00 |
|  | 30 | 8 | 5 | 0 | 1 | 0 | 1 | 7 | 87.50 |
|  | 40 | 1 | 1 | 0 | 0 | 0 | 0 | 1 | 100.00 |
| 2013 | 15 | 34 | 5 | 1 | 0 | 0 | 0 | 6 | 17.65 |
|  | 20 | 20 | 2 | 1 | 5 | 0 | 0 | 8 | 40.00 |
|  | 25 | 0 | 0 | 0 | 0 | 0 | 0 | 0 | — |
